# Prediction and treatment of asthma in preschool children at risk: study design and baseline data of a prospective cohort study in general practice (ARCADE)

**DOI:** 10.1186/1471-2466-9-13

**Published:** 2009-04-15

**Authors:** Karina E van Wonderen, Lonneke B van der Mark, Jacob Mohrs, Ronald B Geskus, Willem M van der Wal, Wim MC van Aalderen, Patrick JE Bindels, Gerben ter Riet

**Affiliations:** 1 Department of General Practice, Academic Medical Center, Amsterdam, the Netherlands; 2 Department of Clinical Epidemiology, Biostatistics and Bioinformatics, Academic Medical Center, Amsterdam, the Netherlands; 3 Pediatric Respiratory Medicine, Emma Children's Hospital – Academic Medical Center, Amsterdam, the Netherlands; 4 Department of General Practice, Erasmus Medical Center, Rotterdam, the Netherlands

## Abstract

**Background:**

Asthma is a difficult diagnosis to establish in preschool children. A few years ago, our group presented a prediction rule for young children at risk for asthma in general practice. Before this prediction rule can safely be used in practice, cross-validation is required. In addition, general practitioners face many therapeutic management decisions in children at risk for asthma. The objectives of the study are: (1) identification of predictors for asthma in preschool children at risk for asthma with the aim of cross-validating an earlier derived prediction rule; (2) compare the effects of different treatment strategies in preschool children.

**Design:**

In this prospective cohort study one to five year old children at risk of developing asthma were selected from general practices. At risk was defined as 'visited the general practitioner with recurrent coughing (≥ 2 visits), wheezing (≥ 1) or shortness of breath (≥ 1) in the previous 12 months'. All children in this prospective cohort study will be followed until the age of six. For our prediction rule, demographic data, data with respect to clinical history and additional tests (specific immunoglobulin E (IgE), fractional exhaled nitric oxide (FENO), peak expiratory flow (PEF)) are collected. History of airway specific medication use, symptom severity and health-related quality of life (QoL) are collected to estimate the effect of different treatment intensities (as expressed in GINA levels) using recently developed statistical techniques. In total, 1,938 children at risk of asthma were selected from general practice and 771 children (40%) were enrolled. At the time of writing, follow-up for all 5-year olds and the majority of the 4-year olds is complete. The total and specific IgE measurements at baseline were carried out by 87% of the children. Response rates to the repeated questionnaires varied from 93% at baseline to 73% after 18 months follow-up; 89% and 87% performed PEF and FENO measurements, respectively.

**Discussion:**

In this study a prediction rule for asthma in young children, to be used in (general) practice, will be cross-validated. Our study will also provide more insight in the effect of treatment of asthma in preschool children.

## Background

Asthma is the most prevalent chronic illness in children. It is an inflammatory disorder of the airways and is strongly associated with airway hyperresponsiveness and symptoms like wheezing, shortness of breath, and coughing [1,2]. Potential predictors for asthma in childhood or later in life have been studied widely. Predictors that have already been identified include environmental factors; i.e. exposure to allergens [3-6], tobacco smoke [7,8] , respiratory (viral) infections [9-11], and diet (particularly breastfeeding) [12]. But also 'non environmental' factors such as sex [13,14] and obesity [15,16] are predictors for asthma. It is thought that early identification of children at high risk for asthma may improve their management resulting in fewer respiratory symptoms, exacerbations and emergency medical visits while improving their quality of life (QoL) and preventing loss of lung function and airway remodelling over time [17-20].

A few studies have derived prediction rules to predict asthma later in life [21-23]. Perhaps the most well known prediction rule (clinical asthma-risk index) was developed in the Tucson Children's Respiratory Study by Castro-Rodriguez et al. [22]. This prediction rule was constructed in preschool children from the general population with symptoms of frequent wheezing. Although this prediction rule is helpful to identify children at high risk of (developing) asthma later in life in the general population, it cannot automatically be used in general practice. Factors that determine which children will visit the general practitioner (GP) are not incorporated in the Tucson prediction rule, influencing the strength of the components in the rule. Also a diagnosis of asthma later in life was based on surveys which is a less objective measure compared to a clinical outcome based on spirometry and hyperresponsiveness.

Therefore, Eysink et al. [23] presented a prediction rule for general practice with factors that determine which children will visit the GP. This prediction rule also used an objective outcome measure of asthma at age six; i.e. a combination of current symptoms (complaints of wheezing and/or shortness of breath and/or recurrent coughing) and/or use of β_2 _agonists and/or inhaled corticosteroids during the previous 12 months in combination with airway hyperresponsiveness to methacholine (PC_20 _FEV_1 _≤ 8.0 mg/ml, or > 10% increase in FEV_1 _after rapid acting β_2 _agonists (salbutamol) inhalation if baseline airflow obstruction precluded the methacholine challenge). The Eysink prediction rule was based on age at presentation, wheezing, family history of allergy for pollen, and specific immunoglobulin E (IgE) to house dust mite, cat and dog dander. Although the asthma probability varied from 1.3% to 94.5% with a bootstrapped area under the curve (AUC) between 0.78 to 0.92, it is essential that the rule is validated prospectively on a separate population before use in practice [24]. Clinical prediction rules typically demonstrate reduced performance in a new patient population because they are optimally modeled to the original data set. In the present study Eysink's existing prediction rule will be cross-validated.

Although prediction of asthma in preschool children is important with a view to prevention, the GP also faces therapeutic management decisions in these children at risk of developing asthma. Currently, treatment intensity is categorized according to the international Global Initiative for Asthma guidelines (GINA) [25]. However, according to GINA, available literature on treatment of asthma in preschool children precludes detailed treatment recommendations. Moreover, randomized trials (RCTs) in these young children in primary care are not forthcoming. Therefore, we will determine the effect of different treatment intensities (GINA levels) on symptoms and QoL in preschool children at risk for asthma in a prospective cohort setting. In our population-based prospective cohort study, we will compare the effects of no treatment and the different GINA treatment intensity levels most commonly used by Dutch GPs. The prescription histories will shed an indirect light on treatment adherence (frequency of repeat prescriptions), but the main strength of our approach is that we learn about real-life effects of what physicians prescribe/advise, incorporating real-life adherence levels.

The AiRway Complaints and Asthma DEvelopment (ARCADE) prospective cohort study has two main objectives. First, we will cross-validate the prediction rule of Eysink et al. Second, the effect of different treatment strategies on symptoms and QoL in preschool children will be compared.

This article reviews the study design and baseline data of the children in this general practice based study.

## Methods and design

The ARCADE study is an ongoing multicenter, prospective cohort study which started in 2004 and will end in 2011, when all enrolled children have reached the age of six years. Additional file 1 shows the time frame of the study, including details of the type of contacts with the study population in the various phases of the study. The study was approved by the Central Committee on Research Involving Human Subjects (CCMO/P04.0098C).

### Enrollment of children and time frame

In three areas in The Netherlands, one to five year old children at risk to develop asthma were selected from 14 general practices. Children at risk for asthma were defined as 'visited the general practitioner with recurrent coughing (≥ 2 visits), wheezing (≥ 1) or shortness of breath (≥ 1) in the previous 12 months'.

**Figure 1:**
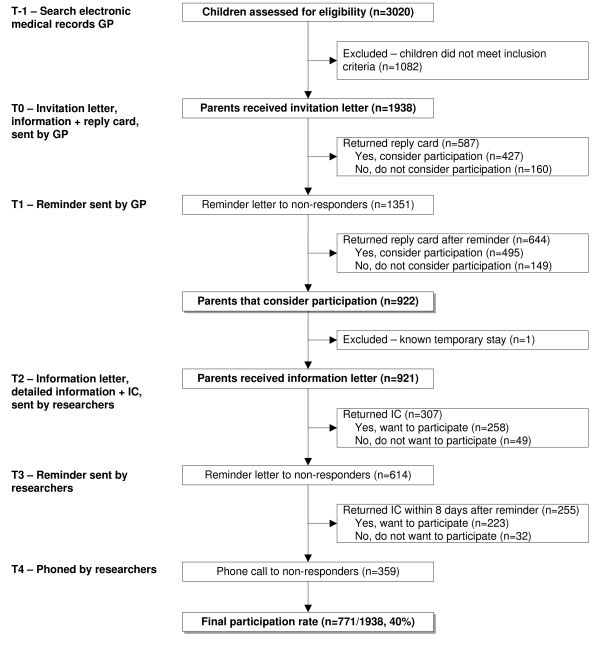
**Flowchart of children in the study**. IC: Informed consent GP: General practitioner.

Figure 1 shows the flow of patients through the study. Briefly, Parents of eligible children received mailed information (including a reply card) about the study from their GP (stage 1). On the reply card the parent(s) could indicate whether they considered participation in ARCADE. A reminder letter was sent to parents who had not returned the reply card, 7 days after the mailing. All parents that indicated considering participation in the study received detailed written information (with an informed consent form) from the researchers (stage 2). After seven days a reminder was sent to all parents who had not returned the informed consent form. Subsequently, all parents who had not responded to the reminder were approached by telephone. Parents of children who returned a signed informed consent form were included in ARCADE.

### Validation and updating of asthma prediction rule

#### Sample size calculation

The final prediction rule will contain up to 5–10 variables, taking into account practical efficiency. A widely accepted rule is that for each variable about 10 cases are required to prevent over-fitting of the model (Events Per Variable rule) [26]. This implies that 100 cases of asthma are needed to model these 10 variables. We expect the prevalence of asthma in the ARCADE cohort to be about 15%. With the inclusion of 771 children in the study, we may screen 11 variables. This prediction rule will be validated with the existing one of Eysink et al., as is recommended in the pertinent literature [24].

#### Measurements (Additional file 1)

##### Questionnaire background & symptoms

The parents of the children, annually, receive a questionnaire on (changes in) housing conditions, family history of allergy, asthma and eczema, presence of pets, breastfeeding, and asthma-related symptoms until the children reach the age of 6. Information about wheezing, rhinitis, eczema, cough, and phlegm is obtained by the Core Questionnaire of the International Study of Asthma and Allergy in Children (ISAAC) [27].

##### Allergy

Total immunoglobulin E (total IgE) and specific immunoglobulin E (specific IgE) directed against house dust mite, cat and dog dander is determined by radioallergosorbent test (RAST) at baseline [22,28]. Children under 4 who tested negative at baseline will be retested at the age of 4, to assess the predictive value of sero-conversion. A convenient method (finger prick) for sampling blood for analyses of total and specific IgE is used [28].

IgE positivity to house dust mite, cat and/or dog dander is defined as > 0.35 kU/l.

##### Inflammatory markers

Fractional Exhaled Nitric Oxide (FENO) is measured in the hospital or general practice at age 5 using an offline technique. Exhaled air is collected in a NO-impermeable Mylar balloon (ABC balloons, Zeist, The Netherlands). All balloons are analyzed in a NO-analyzer (Aerocrine AB; Sweden) within a time period of 6–8 hours after taking the samples [29].

##### Spirometry at age 5

Peak expiratory flow (PEF) is measured twice daily over a period of 14 days at age 5. PEF is performed on a One Flow FVC Memo (Clement Clark International, Essex, United Kingdom) by the children in their home environment, after a personal demonstration by a research assistant. The One Flow FVC Memo measures and stores the PEF automatically. Thus, errors due to incorrect reading and registration are prevented [30].

#### Outcome (asthma at age 6)

Asthma is defined as a combination of current symptoms (complaints of wheezing and/or shortness of breath and/or recurrent coughing) and/or use of asthma medication (β_2 _agonists and/or inhaled corticosteroids) during the previous 12 months in combination with airway hyperresponsiveness to methacholine. Airway hyperresponsiveness is defined as PC_20 _FEV_1 _≤ 8.0 mg/ml, or > 10% increase in FEV_1 _after rapid acting beta-2-agonists (salbutamol) inhalation if baseline airflow obstruction precluded a methacholine challenge [31].

Spirometry is obtained using a Pulmoassist 2 spirometer (Jaeger, Würzburg, Germany). Bronchial responsiveness to increasing doses of methacholine (PC_20_) is measured with a gauged DeVilbiss 646 nebulizer (DeVilbiss, Somerset, MA, USA) with an output of 0.13 ml/min according to the modified method of Cockroft et al. [32]. Children on asthma-medication will withheld all bronchodilators 48 hours before the test. In case of shortness of breath children receive ongoing rapid acting beta-2-agonists up to 8 hours before the test.

#### Statistical analysis prediction rule

At the time of writing, a failsafe model selection strategy on which all statisticians and other experts in diagnostic and predictive modeling agree does not appear to exist. However, in his book 'Regression modeling strategies (2001)' [33] , Harrell made some general proposals for researchers to tailor to the specific circumstances. We intend to use penalized logistic regression, and Tibshirani's *lasso *(least absolute shrinkage and selection operator) [34] in particular to combine the requirements of counteracting overoptimism (shrinkage of regression coefficients) while leaving the opportunity that some coefficients are set to zero, which serves the requirement of a parsimonious model.

Bootstrapping will be used to estimate the penalization coefficient [34]. As a form of sensitivity analysis, we shall also explore Sauerbrei and Schumacher's bootstrapped stepwise regression [35] (p-entry = 0.15; p-remove = 0.20; predictor retained if selected in >70% of bootstrap samples; method = forward) to see how well these approaches concur. We will avoid univariable preselection of predictors. The linearity assumption will be checked for all continuous predictors. The final model will be bootstrapped.

Discrimination of the model will be visualized in high resolution histograms and summarized as 5th, 10th, 25th 50th, 75th, 90th, and 95th centiles of these, Brier score and the area under the receiver operating characteristics curve (ROC) with 95% confidence intervals (overall discrimination) [36].

Using the regression coefficients of the independent diagnostic indicators, an easy to use, multivariable diagnostic rule (asthma prediction rule) will be derived, consisting of relevant tests and their diagnostic values.

### Effect of different treatment intensities

#### Continuous registration of treatment by GP

As children reach the age of 6 and follow-up ends, the medication prescription histories within the ARCADE period will be read from the GPs' electronic medical records and classified into one of the Global Initiative for Asthma Guidelines [25] levels for treatment. As medication histories may change over time, they will be treated as time-varying exposures.

#### Continuous registration of respiratory symptoms by GP

The GPs will score nine common airway symptoms (such as symptoms of coughing, wheezing and shortness of breath) in a standardized way 'A9-form'. These items are scored each time a child participating in ARCADE visits the GP with airway complaints. Registration will be carried out in an electronic way (a pop-up menu).

#### QoL measurements

Every 6 months until the children reach the age of 6, the parents of the children receive a (health related) QoL questionnaire (PAQLQ/CHQ) [37,38].

#### Measurements of outcome

The main outcome measures are mean severity level (number of symptoms scored as positive) and health-related QoL. These measures will be calculated at specific time-points (e.g. the effect at 12 months after initiation) and longitudinally over time.

#### Statistical analyses medication strategies

The causal effect of interest is that of treatment strategies (GINA classified) on complaint severity and (health-related) QoL. However, complaint intensity and QoL act as time-dependent confounder and intermediary factor at the same time. Complaint intensity acts as time-dependent confounder because children with more severe complaints are more likely to receive more aggressive treatment, and present complaint level may predict future complaint intensity. It acts as intermediate variable because the treatment they receive may change complaints and thus complaints are in the intermediate pathway to QoL. The same holds true for QoL and complaint intensity and QoL may also influence each other. Analysis of the treatment effect using standard methods (such as Cox regression), adjusting for the confounding by indication by including both variables in the model will then cause bias [39]. In particular, the indirect effect of treatment through complaints will be lost by conditioning on complaint level.

We will use more recently developed methods for causal inference from observational data, such as marginal structural models (MSM) [40-49]. Marginal structural models use the detailed information on each child to predict treatment allocation (more severe complaints on average trigger more intense treatment levels) and adjust for it appropriately. This yields estimates of the causal effect of treatment comparable to that obtained from a RCT, assuming that all important confounders are measured and correctly adjusted for.

We plan to study the effects of fixed treatment levels as well as the effects of dynamic treatment regimes. The latter analysis emulates a RCT in which one treatment arm may, for example, receive the following (dynamic) regime: immediately step up one GINA level if complaints are not fully controlled (as measured by our nine clinical items). This may be compared to a regime stipulating that the number of levels to be stepped up must depend on the degree of non-control (partly uncontrolled – uncontrolled – exacerbation) [50-53].

## Discussion

### Results

#### Patient selection and participation

For the ARCADE cohort, 3020 children were selected from 14 general practices in three cities in The Netherlands between October 2004 and July 2006 (figure 1). On average, 138 children (range 44 to 426) per practice were identified from the electronic medical records of the GPs by using search terms related to coughing, wheezing and shortness of breath. One researcher (KvW) verified the computer search by checking case notes and a total of 1,938 children were defined as 'at risk' for developing asthma and deemed suitable for ARCADE by their GP. Reasons for non-approval included (important) comorbidity, known temporary stay in the region or parents unable to read or understand Dutch or English. Of all children, 921 parents considered participation – returned the reply card, wanted to receive detailed information and an informed consent (IC) form. In total 771 parents were enrolled in the cohort study. The overall participation rate was 40% (771/1938). The children of parents that were enrolled in the study did not differ on age, sex and symptoms at onset to the children that were not enrolled (Table 1).

**Table 1:** Characteristics of children enrolled in the study

	**Enrolled in the study (%)** **(n = 771)**	**Not enrolled in the study (%)** **(n = 1167)**
**Sex**		
Male	432 (56.0)	630 (54.0)
Female	339 (44.0)	538 (46.1)
**Age at onset**		
1	267 (34.6)	349 (29.9)
2	220 (28.5)	343 (29.4)
3	127 (16.5)	227 (19.5)
4	121 (15.7)	203 (17.4)
5	36 (4.7)	45 (3.9)
**Symptoms at onset**		
**One respiratory symptom**	**518 (67.2)**	**897 (76.9)**
Cough (2×)	420 (54.5)	763 (65.4)
Wheeze	57 (7.4)	83 (7.1)
Shortness of breath	41 (5.3)	51 (4.4)
		
**Two respiratory symptoms**	**195 (25.3)**	**221 (18.9)**
Wheeze and cough	121 (15.7)	150 (12.9)
Shortness of breath and cough	55 (7.1)	55 (4.7)
Shortness of breath and wheeze	19 (2.5)	16 (1.4)
		
**Three respiratory symptoms**	**58 (7.5)**	**49 (4.2)**
Shortness of breath, wheeze and cough	58 (7.5)	49 (4.2)

Numbers are n (percentages in parentheses)

#### Compliance with the study protocol (after 18 months of follow-up)

As the assessment schedule shows (Table 2), ARCADE collects data on covariates at baseline and annually using questionnaires (e.g. ISAAC and QoL). The response rates to these repeated questionnaires varied from 93% at baseline to 74% after 18 months of follow-up. After 18 months of follow-up, 49 parents indicated that they did not want to participate any longer. The reasons why parents indicated to stop included lack of time, lost interest in the study or child did not have airway complaints anymore.

**Table 2:** Details of follow-up of the first 18 months of the cohort study

	**Number of children invited to perform measurement**	**Number of children that performed measurement**
**Questionnaire**		
Baseline – symptoms and QoL^a^	771	718 (93.1)
Follow-up at 6 months – QoL^a^	755	643 (85.2)
Follow-up at 12 months – symptoms and QoL^a^	707	551 (77.9)
Follow-up at 18 months – QoL^a^	610	450 (73.8)
		
**Total and specific IgE measurement **^**b**^	771	670 (86.9)
		
**NO-measurements **^**c**^	131	114 (87.0)
		
**PEF-measurements **^**d**^	131	117 (89.3)

Numbers are n (percentages in parentheses)

^a ^QoL: Quality of Life measurement

^b ^IgE: Immunoglobulin E

^c ^NO: Nitric oxide measurement

^d ^PEF: Peak Flow measurement

Total serum IgE and specific serum IgE, performed at baseline, resulted in a response rate of 87%. After a follow-up of 18 months, 131 5-year old children have been invited to perform a peak flow measurement and a FENO-measurement. Data were complete for 89% and 87%, respectively.

#### Outcome (asthma at age 6)

After 18 months of follow-up, 32 children in the ARCADE cohort reached the age of 6, of whom seven did not have respiratory symptoms nor used asthma medication in the previous 12 months. These children were defined as not having asthma. The remaining 25 children (77%) had complaints of wheezing and/or shortness of breath and/or recurrent coughing and/or had used asthma medication in the previous 12 months. These children were all invited for a methacholine challenge test to confirm or refute a diagnosis of asthma.

### Discussion

The ARCADE study started two years ago and is an ongoing multicenter, prospective cohort study in which a prediction rule for 1 to 5 year old children at risk for developing asthma will be constructed. Also, the effects of frequently used 'real-world' treatment strategies on asthma severity in young children will be estimated.

Children were eligible for ARCADE if they were at risk of developing asthma; i.e. they visited their GP with complaints of coughing, wheezing and/or shortness of breath. We were able to select 1,938 children from the electronic medical records of the GPs and recruited 40% of those identified as at risk. Participation rates increased with the number of respiratory symptoms in the previous year. In the coming years all children will be followed until the age of 6.

### Choices of methods in our study

#### Validation and updating of asthma prediction rule

For the construction of the prediction rule we collect data easy to obtain in general practice. Therefore we chose demographic data, data with respect to clinical history and additional tests that can easily be performed in general practice; i.e. total and specific IgE, FENO, and PEF. Measuring FENO using an offline technique is time consuming and expensive [29] however currently FENO can be measured with the 'NIOX mino' [54] , a small device that provides quick and easy FENO data. Other additional tests that could be performed in young children are the interrupter technique (Rint) and exhaled breath condensate (EBC). Although, the (additional) value of these tests is at present studied on a large scale, these measurements cannot be performed in general practice and were therefore not included in our study.

Several studies have postulated that early use of inhaled corticosteroids could prevent the onset of asthma and suggest over-treatment at a young age since inhaled corticosteroids appears to be effective in reducing symptoms in high-risk young children with frequent wheezing [17-20]. However, recent large RCT studies of Bisgaard et al. [55] and Guilbert et al. [56] showed that it seemed unlikely that early treatment prevents asthma. Therefore, we determined not to include the use of asthma medication such as β_2 _agonists and inhaled corticosteroids during the study period as a variable in our prediction rule.

#### Effect of different treatment intensities

We will compare the effects of no treatment and the different GINA treatment intensity levels most commonly used by Dutch GPs in a prospective cohort study. Although one might prefer a rigorous RCT, even these do not always shed light on all aspects that are relevant for treatment decisions. This is so because many RCTs are atypical in several aspects: atypical patients (restrictive clinical domain), atypical levels of compliance (good monitoring), atypical quality and compliance of concomitant treatment (strict protocols), and, finally, not all relevant treatment strategies may be compared (companies prefer placebo-controls over head-to-head comparisons although the latter are often far more relevant). Our population-based prospective cohort study avoids all these atypical features. We lack the safety net of randomization. We are confident however, that important confounders are measured reliably in our study.

### Problems

During follow-up a number of parents indicated that they did not want to participate any longer. Reasons to stop participating in the study included lack of time, loss of interest in the study or disappearance of airway complaints. Dropping out from the study due to reasons that affect the outcome of asthma at age six is called informative censoring and needs to be corrected for. To estimate the effect of treatment on an outcome, the sample is weighted to correct for informative censoring. This is done by estimating for each individual, at each time point, the probability of her observed censoring history given her observed covariate history. The weights are the inverse of these probabilities. By weighting in this manner, individuals with a "rare" censoring history (for example unhealthy, but late censoring) receive a larger weight, whereas individuals with a more common censoring history (for example unhealthy and early censoring) are assigned a smaller weight. Via weighting, the sample will resemble a sample in which no informative censoring is present.

## Conclusion

The ARCADE study is an ongoing multicenter, prospective cohort study in which an existing prediction rule for 1 to 5 year old children at risk of developing asthma, will be validated and updated when needed. Also, the effect of different treatment strategies in young pre-school children, carried out by the GP, will be compared. This will provide more insight in treatment of asthma in young children since available literature on treatment of asthma in pre-school children precludes detailed treatment recommendations.

## Abbreviations

ARCADE study: AiRway Complaints and Asthma Development study; AUC: Area under the curve; CCMO: Central Committee on Research Involving Human Subjects; FENO: Fractional exhaled nitric oxide; FEV_1_: Forced expiratory volume in one second; GINA: Global INitiative for Asthma guidelines; GP: General practitioner; IC: Informed Consent; IgE: Immunoglobulin E; ISAAC: International Study of Asthma and Allergy in Children; MSM: Marginal structural models; PC_20_: Concentration of methacholine that induced a 20% fall in FEV_1_; PEF: Peak expiratory flow; QoL: Quality of Life; RCT: Randomized controlled trial; ROC: Receiver operating characteristics curve.

## Competing interests

The authors declare that they have no competing interests.

## Authors' contributions

KvW participated in working out the protocol, wrote the first draft of this article and will analyse the data. GtR participated in the design of the study, coordinates the project and helped to draft the article. WvA, RG, LvdM, JM, WvdW, PB participated in the design of the study and JM also helped to collect the data. PB commented on article concepts, is the project leader of the study, and supervised the project.

All authors have read and approved the final manuscript.

## Pre-publication history

The pre-publication history for this paper can be accessed here:



## Supplementary Material

Additional file 1 **Table s1**. The AiRways Complaints and Asthma Development (ARCADE) time frame Click here for file
