# Supplementary material for: Prediction and treatment of asthma in preschool children at risk: study design and baseline data of a prospective cohort study in general practice (ARCADE)
Source: BMC Pulm Med. 2009 Apr 15;9:13. doi: 10.1186/1471-2466-9-13 (PMC2678979; doi:10.1186/1471-2466-9-13)
Supplement: Additional file 1 — Table s1. The AiRways Complaints and Asthma Development (ARCADE) time frame [file 1471-2466-9-13-S1.doc]

| **Age**  **(yrs)** | **Objective** | **Baseline** | | **0.5**  **yr** | | **1**  **yr** | | **1.5**  **yrs** | | **2**  **yrs** | | **2.5**  **yrs** | | **3**  **yrs** | | | **3.5**  **yrs** | | | **4**  **yrs** | **4.5**  **yrs** | | **5**  **yrs** | | |  |
| --- | --- | --- | --- | --- | --- | --- | --- | --- | --- | --- | --- | --- | --- | --- | --- | --- | --- | --- | --- | --- | --- | --- | --- | --- | --- | --- |
| 1 | **Prediction**  **rule**  **Treatment**  **effect** | questionnaire  total IgE^a^  specific IgE^a^  QoL^b^  — — — — — — — — — — — — — — — — — — *Registration of symptoms & treatment by GP*  — — — — — — — — — — — — — — — — — | | QoL^b^ | | questionnaire  QoL^b^ | | QoL^b^ | | questionnaire  QoL^b^ | | QoL^b^ | | questionnaire  QoL^b^ | | | QoL^b^ | | | questionnaire  NO^c^; PEF^d^  QoL^b^ | QoL^b^ | | questionnaire  spirometry +  MCT^e^  QoL^b^ | | |  |
| 2 | **Prediction**  **rule**  **Treatment**  **effect** | questionnaire;  total IgE^a^  specific IgE^a^  QoL^b^    — — — — — — — — — — — — — *Registration of symptoms & treatment by GP*  — — — — — — — — — — — — — | | QoL^b^ | | questionnaire  QoL^b^ | | QoL^b^ | | questionnaire  QoL^b^ | | QoL^b^ | | questionnaire  NO^c^; PEF^d^  QoL^b^ | | | QoL^b^ | | | questionnaire  spirometry +  MCT^e^  QoL^b^ |  | |  | | |  |
|  | |  |
| 3 | **Prediction**  **rule**  **Treatment**      — — — — — — — — *Registration of symptoms & treatment by GP*  — — — — — — — — —  **Effect** | questionnaire  total IgE^a^  specific IgE^a^  QoL^b^ | | QoL^b^ | | questionnaire  QoL^b^ | | QoL^b^ | | questionnaire  NO^c^; PEF^d^  QoL^b^ | | QoL^b^ | | questionnaire  spirometry +  MCT^e^  QoL^b^ | | |  | | |  |  | |  | | |  |
| 4 | **Prediction**  **rule**  **Treatment**  **effect** | questionnaire  total IgE^a^  specific IgE^a^  QoL^b^    — — — — *Registration of symptoms & treatment by GP*  — — — — | | QoL^b^ | | questionnaire  NO^c^; PEF^d^  QoL^b^ | | QoL^b^ | | questionnaire  spirometry +  MCT^e^  QoL^b^ | |  | |  | |  | |  |  | | | | | |  | |
|  | | | |
| 5 | **Prediction**  **rule**  **Treatment**  **effect** | | questionnaire  total IgE^a^  specific IgE^a^  NO^c^; PEF^d^  QoL^b^  *Registration of symptoms & treatment by GP* | | QoL^b^ | | questionnaire;  spirometry +  MCT^e^  QoL^b^ | |  | |  | |  | |  | | | | | | |  | |  | | |

^a^ IgE: Immunoglobulin E

^b^ QoL: Quality of Life measurement

^c^ NO: Nitric oxide measurement

^d^ PEF: Peak Flow measurement

^e^ MCT: Methacholine Challenge Test
